# Autonomous drone hunter operating by deep learning and all-onboard computations in GPS-denied environments

**DOI:** 10.1371/journal.pone.0225092

**Published:** 2019-11-18

**Authors:** Philippe Martin Wyder, Yan-Song Chen, Adrian J. Lasrado, Rafael J. Pelles, Robert Kwiatkowski, Edith O. A. Comas, Richard Kennedy, Arjun Mangla, Zixi Huang, Xiaotian Hu, Zhiyao Xiong, Tomer Aharoni, Tzu-Chan Chuang, Hod Lipson

**Affiliations:** 1 Department of Mechanical Engineering, Columbia University, New York, New York, United States of America; 2 Department of Computer Science, Columbia University, New York, New York, United States of America; 3 Department of Electrical Engineering, Columbia University, New York, New York, United States of America; Universiti Sains Malaysia, MALAYSIA

## Abstract

This paper proposes a UAV platform that autonomously detects, hunts, and takes down other small UAVs in GPS-denied environments. The platform detects, tracks, and follows another drone within its sensor range using a pre-trained machine learning model. We collect and generate a 58,647-image dataset and use it to train a Tiny YOLO detection algorithm. This algorithm combined with a simple visual-servoing approach was validated on a physical platform. Our platform was able to successfully track and follow a target drone at an estimated speed of 1.5 m/s. Performance was limited by the detection algorithm’s 77% accuracy in cluttered environments and the frame rate of eight frames per second along with the field of view of the camera.

## Introduction

The number of unmanned aerial vehicles (UAVs) is growing rapidly. In the US alone, approximately 3.55 million small UAVs are expected to be deployed for consumer use by 2020 [1]. Accordingly, the misuse risk of UAVs is also increasing. Reports of drones flying too close to airports [2–4]; privacy violations of civilians [5,6]; interfering with military or law-enforcement operations [7,8]; or used for other malicious purposes [9,10] are becoming more frequent. Furthermore, as commercially available drone technology evolves, drone-related crimes can become more sophisticated. While we wait for legislative efforts to catch up with technological advancements, we anticipate a growing need for technological solutions that can autonomously defend against malicious UAVs.

We propose an autonomous hunting drone—a self-contained UAV defense system that can chase and neutralize another drone in a GPS-denied environment while performing all computations on board. During project conceptualization, we could not find a suitable drone platform with a powerful enough onboard computer, so we decided to custom build our research platform. Most of the current UAVs operating in GPS-denied environments are vision based, combining optical flow sensors and visual inertial odometry (VIO) with sonar and/or LIDAR for distance measurements [11–13]. Following this trend, we adopted a vision-based localization system.

Autonomously hunting and neutralizing a rogue drone is a complex task requiring a series of steps: (1) initialization and preflight checks, (2) takeoff and target search, (3) target tracking and hunting, and (4) a takedown procedure. The main challenge is the hunting stage since autonomous takeoff is achievable once basic localization is established. Selecting appropriate sensors and implementing a search pattern solves the target search. Takedown is trivial in a close-proximity situation, as most drones are fragile and easily disabled by propeller restriction or electronic interference. Thus, our research focuses on the hunting stage. The goal is to approach the target close enough to engage it.

## Related work

The technologies involved in building a successful drone hunter have been subject to research for years: vision-based localization and tracking, object detection, GPS-denied navigation, obstacle avoidance, friend or foe classification, and UAV dynamics and control. We will focus on two kinds of related works. First, we will address the commercial anti-drone solutions available today. Second, we address selected papers that show interesting approaches to UAV tracking.

The state-of-the-art technologies that remove drones from airspaces can be classified into three groups: signal jamming [14]; propeller restriction (for example, by means of a net) [15,16]; and aerial takedown [17,18]. However, none of these existing systems are an optimal solution for drone hunting in GPS-denied environments. For example, protecting a military convoy from an attack by autonomous drones that do not rely on GPS is likely to fail with current tools. Modules such as PX4Flow and sophisticated companion computers have improved the accessibility of GPS-denied localization and navigation on customized drone platforms [19–21], allowing advanced UAVs to operate even when the GPS and radio signals are jammed. In the presence of these new modules, jamming-based solutions such as the Battelle Drone Defender are rendered ineffective. Although still effective in the absence of a downlink, net guns such as NetGun X1 and Skywall 100 require a skilled operator and are limited in range. A solution most similar to our approach is the Airspace Interceptor, a large hexacopter equipped with a net gun that apprehends a previously selected target[18]. However, instead of being self-contained and agile, the Airspace Interceptor is the part of a holistic drone defense system that is operated from a ground-station trailer and relies on both onboard and off-board sensors. A very unique approach, using eagles to hunt and apprehend UAVs, was explored by the Dutch police and later adopted by the French army[22,23]. Although this approach is innovative, the operator can lose control, endangering birds and bystanders.

UAV detection and tracking is not a new research topic. For example, Opromolla et al. proposed a UAV detection and tracking approach for cooperative flying applications[24]. In their paper, they present a tracker drone that tracks the target drone and provides it with position updates in case it has little-to-no Global Navigation Satellite System (GNSS) reception. The tracker uses the target’s relative GNSS location to identify regions of interest in the camera frame and optimize its tracking precision. The authors take full advantage of the benefits of cooperative flight. The complexity of their implementation, even without the challenges added by adversarial UAV flight and a GPS-denied environment, highlights just how complex implementing an autonomous hunting drone can be.

In “UAV Based Tracking and Recognition,” Xiang et al. present a UAV-based tracking method that leverages a gimbal to improve target tracking [25]. Their system relies on offloading the Geographic Information System database and neural network-based target knowledgebase to a linked ground station, as well as sharing features between the deeper neural network running on the ground station and the simpler neural network onboard the UAV. In addition, responsibility for operating the gimbal is offloaded to a Field Programmable Gate Array (FPGA) chip, helping to ease the load on the onboard Jetson TX1 module. Although their solution is not designed for GPS-denied environments and requires a ground station, it does introduce unique design ideas, such as their use of FPGA technology to operate the gimbal, that could help improve drone hunting in the future.

## Contributions

We present a simple drone hunting platform that self-localizes using VIO via the ZED stereo camera, and runs a visual tracking algorithm on an onboard Jetson TX2 [26]. The algorithm then sends control commands to the PX4-based flight controller [27]. We also generate an annotated dataset of 58,647 images, on which we trained a modified black and white version of the Tiny YOLO detection system [28]. The target drone in a GPS-denied environment was chased by visual servoing without the use of external localization systems, e.g., a motion-capturing system. Our system was implemented on a customized drone platform and tested on a manually piloted target drone in an indoor environment. To the best of our knowledge, no prior research exists on autonomous drone hunting in a GPS-denied environment using a quadcopter platform that performs all computations onboard. Therefore, we aim to bridge this research gap and create a baseline approach for future exploration.

## Structure of the paper

The remainder of this article is organized as follows. In materials and methods section, we describe the data collection and generation process for our drone hunting dataset, discusses our adaptation of the drone-detection algorithm, and finally address our navigation approach for chasing the target drone. The experiments section then presents the experimental results, including our platform design, our hardware and software choices, our classifier tests using both RGB and gray-scale images, and the tests operating our custom drone platform in an indoor environment. In the results section, we present the findings from our experiments, followed by a discussion and a conclusion.

## Materials and methods

### Dataset

To form our drone hunting dataset, we synthetically generated 10,000 images from autonomous drone flying sequences in the AirSim Simulator [29]. We then recorded and manually annotated videos of our target drone while static, moving, and in-flight in multiple environments. The dataset consisted almost exclusively of images of our target drone (with the exception of the synthetic data generated using the Parrot AR drone model in AirSim). In our testing environment, the target drone was chased by an ImmersionRC Vortex 250 UmmaGawd drone equipped with a GoPro Hero5 action camera: collecting authentic drone-to-drone footage. To avoid skewing our dataset by capturing too many identical images at the high frame rate (240 fps) of the GoPro camera, we split the recordings into short sequences, and reduced the frame rate on less erratic flying sequences.

All images were labeled manually using a labeling script. To minimize the labeling time, we skipped the labeling of 10 to 30 frames based on the moving intensity of the drone and the video frame rate and then linearly interpolated the bounding box size and location of the skipped images between any two labeled images.

**Fig 1:**
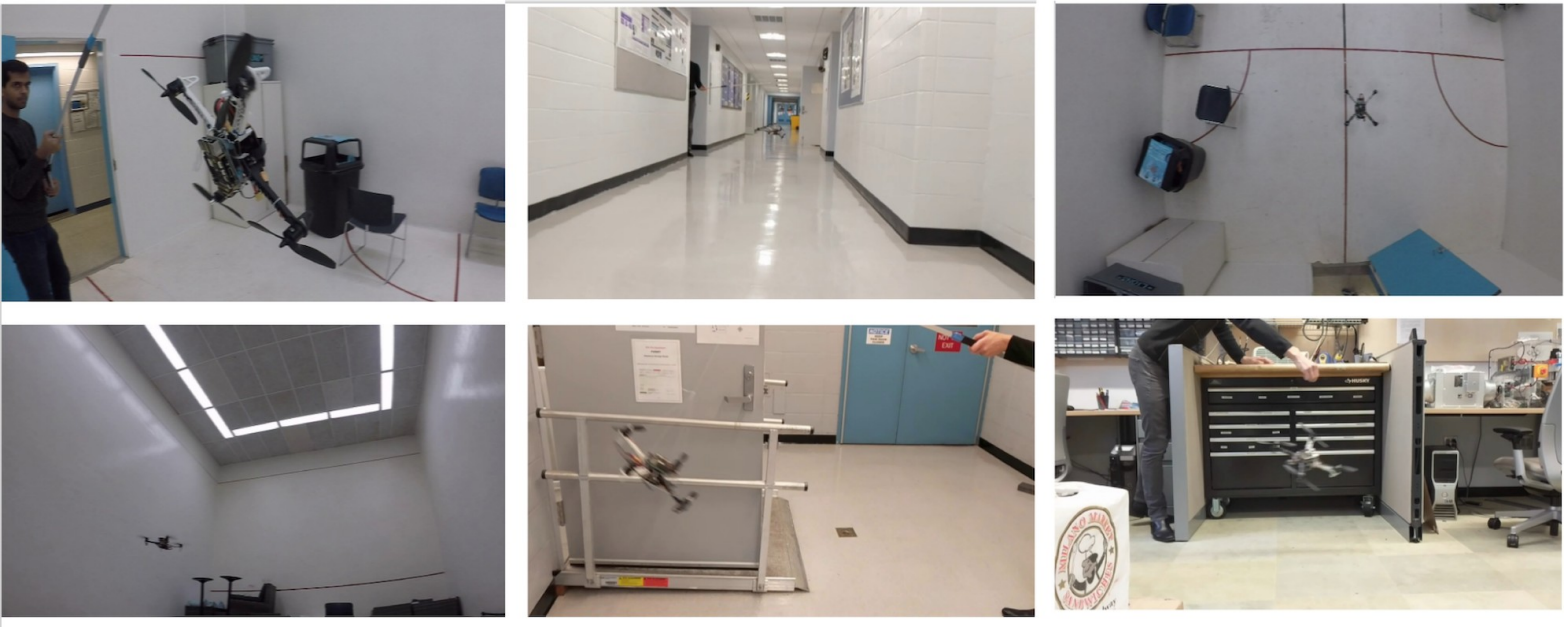
Sample images from our drone detection dataset.

To improve the robustness of our model against motion false positives and motion blur, we included images in different settings with complicated backgrounds (including chairs, fences, and handrails) because our classifier occasionally misinterpreted bars or truss structures as a drone. Moreover, we added motion blur of varying degrees and direction to some of the images. Images were taken in our laboratory, the hallway, a squash court, and outdoors. The drone was captured from all angles. Some sample images are shown in Fig 1. Among the 58,647 images in the current dataset, we randomly selected 90% for the training dataset and retained the remaining 10% as the testing dataset.

### Detection algorithm

#### Drone-tracking approach

Our goal was to detect and track the target drone in the input images captured by the ZED camera and to output the bounding box location and size of the detected drone to the navigation-control system. Although semantic segmentation improves the detection accuracy, it proved to be too resource intensive for our platform. We decided against using a direct, feature-based tracking/detection approach in favor of machine learning methods, since we wanted to create a system that could be retrained on new drone datasets, and that took advantage of the Jetson TX2 GPU cores. In addition, a Haar-like feature-based algorithm has been shown to only run at five frames per second on the Jetson TX2 [30]. Finally, we decided to rely on a bounding box object detection algorithm. To achieve real-time tracking without over-compromising the accuracy, we adopted the Tiny YOLO algorithm.

#### Real-time object detection

Current real-time neural network-based object detectors can be classified into two categories. The first category includes two-stage proposal-driven algorithms such as regional convolutional neural network (R-CNN) [31], Faster-RCNN [32] and FPN [33]. The first stage of these algorithms generates a sparse set of candidate object locations. In the second stage, the CNN classifies each candidate location as one of the foreground classes or as background. The second category embraces one-stage detectors, which are applied over a regular, dense sampling of object locations, scales, and aspect ratios. Single-stage detectors include YOLO [28,34–36]; SSD [37,38]; and RetinaNet [39]. As our network operates on the Jetson TX2 rather than a more powerful graphics processing unit (GPU), we cannot fully exploit the advantages of computationally intensive algorithms. Moreover, as drones can change direction erratically, we sacrificed the detection accuracy to ensure a high frame rate. Although YOLO is not the most accurate among state-of-the art algorithms, it runs significantly faster than other detection methods with comparable accuracy. Among the provided YOLO networks, Tiny YOLO is five times faster than the full-size YOLO network while maintaining an acceptable level of accuracy. Hence, we chose Tiny YOLO as our network architecture.

#### Network architecture

The Tiny YOLO network recognizes 80 classes. We modified the network to detect only the *drone* class. The modified network has nine convolutional layers with 3 × 3 kernel layers and six pooling layers with 2 × 2 kernel layers. The final output of our network is a 13 × 13 × 30 tensor of predictions.

#### Training

Tiny YOLO was trained on a Google Cloud Server equipped with a NVIDIA Tesla P100 GPU. As the pre-trained network, we used YOLO’s Darknet-53 as described in YOLOv3 [36]. The training and testing neural networks were implemented in Darknet, an open source neural network framework written in C and CUDA [40].

### Navigation

The output of our Tiny YOLO detector is the index of the bounding box in the frame. The center of the bounding box relative to the center of the camera frame was used for visual servoing. The visual servoing algorithm operates as follows.

**Fig 2:**
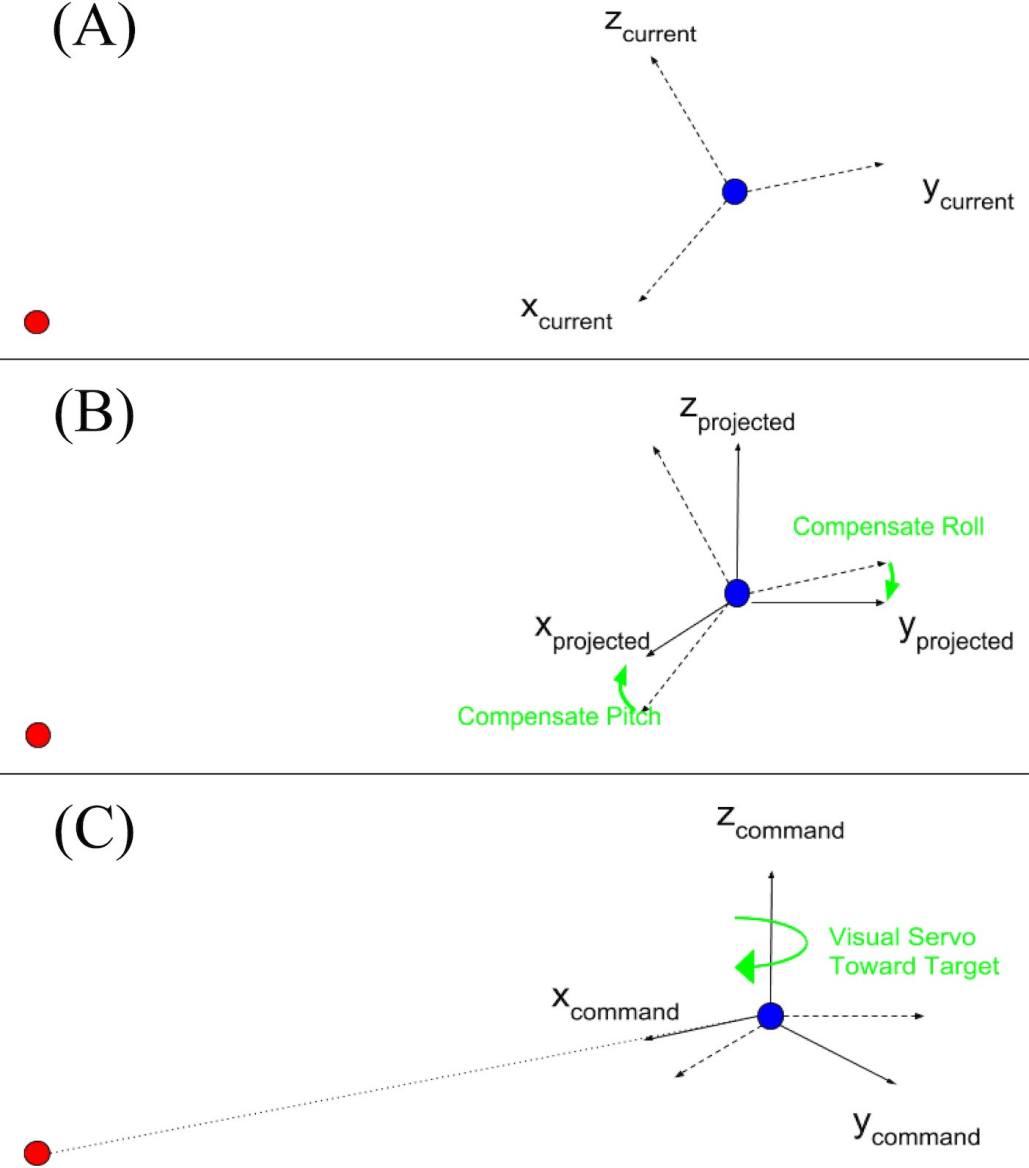
Procedure used to project the current coordinate frame onto the x-y plane for pitch and roll angle compensation. (A) The target (red) is located on the right-hand side of the drone (blue), which is facing in the x-direction. (B) Pitch and roll angle compensation ensures that the command does not flip the drone. (C) The compensated frame is adjusted so that the command is directed toward the target.

- Step 1: Request the location of the target (represented by the bounding box). If a target is detected, move to step 2.
- Step 2: Calculate the direction and step size based on the location of the bounding box relative to the center of the frame.
- Step 3: Stabilize the drone by compensating the roll and pitch angle of the current pose, as shown in Fig 2.
- Step 4: Publish the next set point for the flight controller.

During the hunting process, we adjusted only the *xyz* location and the yaw angle of the hunter approaching the target. To track the target, the flight controller adjusts the roll and pitch angle automatically, moving the hunter drone to the next set point. Unless the drone is moving forward, the roll and pitch remain zero and the drone holds its position.

**Fig 3:**
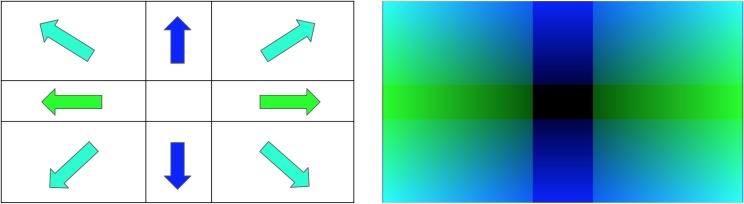
Color representations of the hunter’s next-step responses based on the target’s location in the image frame. Both illustrations are overlaid on the image frames the hunter drone uses to determine its next move. For both methods, the resulting steps combine yaw rotations in radians and ascent or descent in meters. Left: fixed-step hunter response behavior. The center of the bounding box around the target in the camera frame must be located in one of the nine image segments of the 3 x 3 grid. During each control cycle, the hunter responds by taking a fixed step in the direction of the arrow in that image section. Right: proportional hunter response. With this method, the step size and movement direction are determined based on the center of the detected target’s bounding box in the camera image. Here the shades of green and blue represent the left/right and up/down step sizes respectively, with brighter colors denoting larger step sizes and darker colors denoting smaller ones.

We explored two step policies: constant step size and proportional step size. Fig 3 shows the step sizes for both approaches, expressed as a color-coded graph over the image frame where brighter and darker colors denote larger and smaller step sizes respectively, in the direction of the detected target. Green and blue denote horizontal and vertical motion respectively, with intermediate colors denoting proportional mixtures of the two. In our indoor testing environment, we limited the drone’s maximum step size for safety reasons.

**Fig 4:**
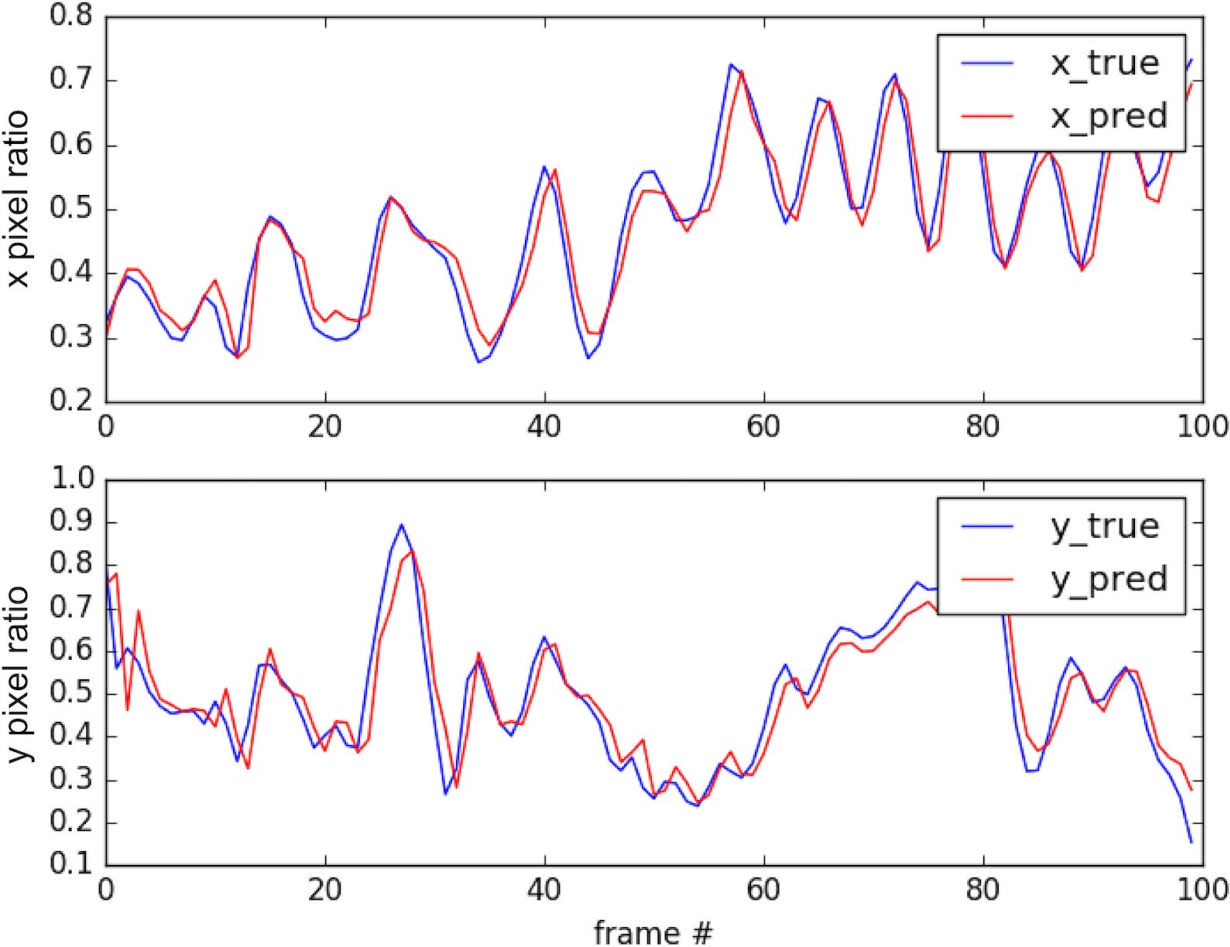
Linearly predicted target location vs. actual target location. Here we compare the trajectory predicted by our regression model (red) with the actual trajectory (blue).

Consequently, when the target drone passed the hunter drone in close proximity or moved erratically, it was lost from the hunter’s camera view. To tackle this problem, we predicted the next location of the target by a linear regression model. The input of the regression model was the centers and sizes of the bounding boxes in the previous *n* steps (size of the history), and the output was the center of the next bounding box. For visualization, Fig 4 shows the *x* and *y* pixel indices on the test dataset predicted with a history size of five. The fitted model was linearly interpolated over the past *n* target locations. The drone’s dynamics limit its acceleration and deceleration rates, so its power constraints limit its ability to change direction. Hence, we assume that the targets trajectory is approximately linear during the observed time interval. Faster, more powerful targets would require higher control-loop frequencies and/or a shorter time intervals for this linear movement assumption to remain valid.

### Experiments

#### Tracking algorithm

We trained two models with different numbers of channels: a three-channel RGB model and a single-channel gray-scale model. To improve the frame rate on Jetson TX2, we trained Tiny YOLO on gray-scale images. Our Tiny YOLO returns a bounding box and confidence rating for each detected drone. However, in our test program, we only present the algorithm with one drone at the time and test the bounding box with the highest confidence. In the evaluations, we compared the speed and accuracy of the two models, as presented in Section IX.

#### Experimental platform

**Fig 5:**
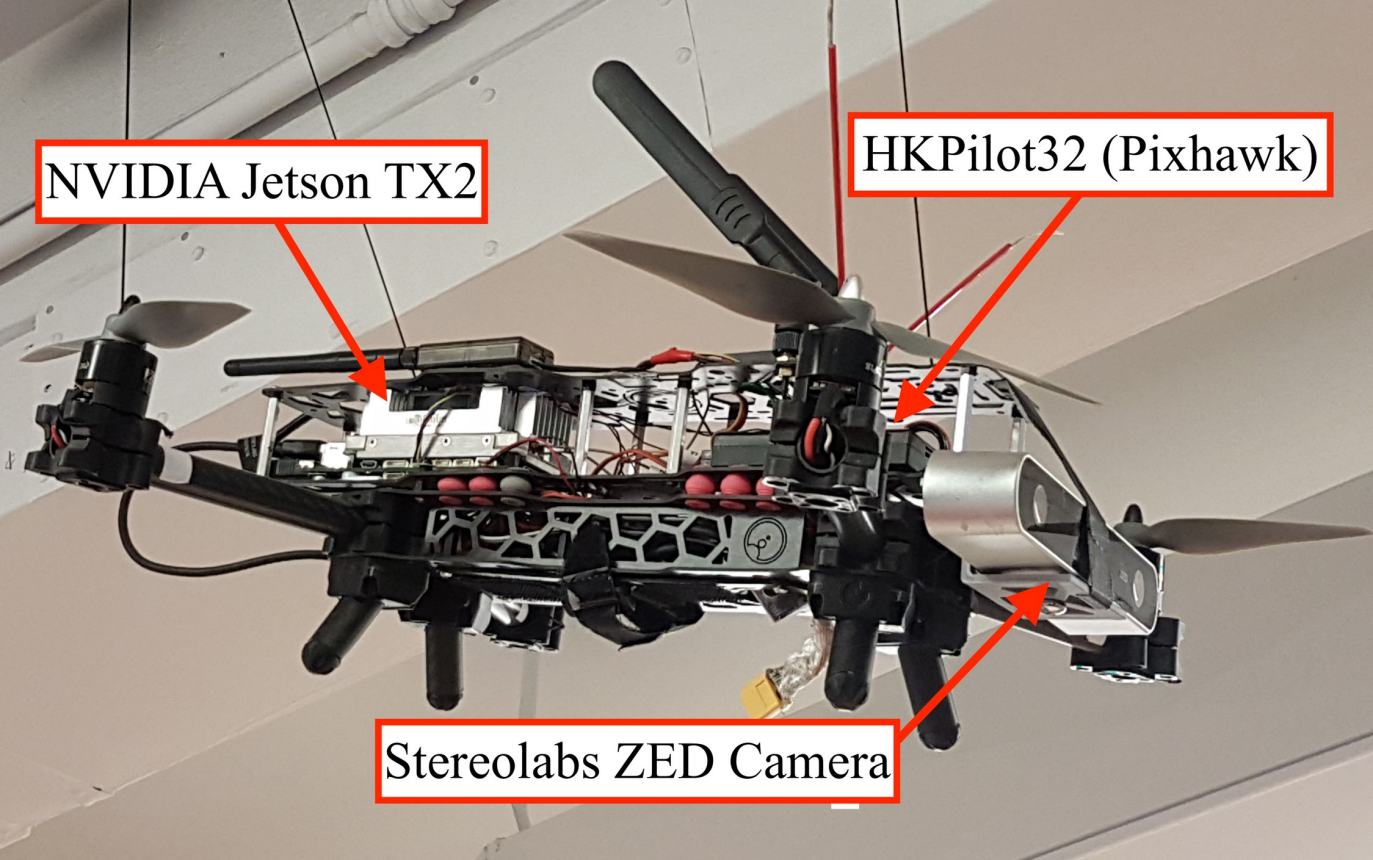
Hunter drone prototype.

**Fig 6:**
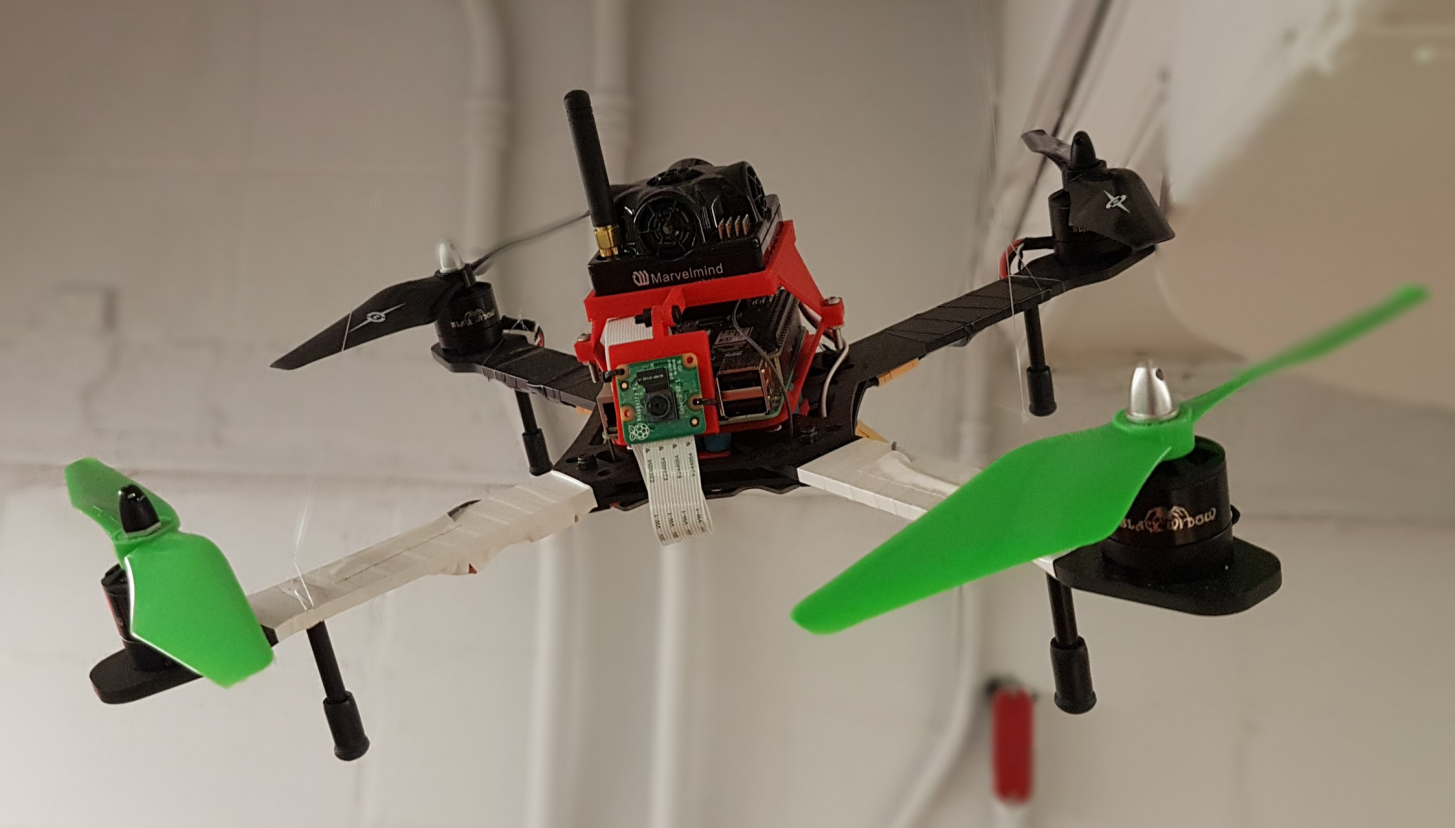
Target drone prototype.

Two drone platforms were designed and built for this project: a hunter drone (Fig 5) and a target drone (Fig 6). The hunter drone is built on the Cinetank MK2 frame and carries an HKPilot32 flight controller running the PX4 LPE firmware, a Jetson TX2 companion computer, and a ZED stereo camera[26,27,41]. It is powered by four Black Widow 2212 1000-kV motors with integrated ESCs, four APC 8x4.5MR propellers, and a 2400-mAh four-cell LiHV battery. The hunter drone weighs 1.3 kg, exerts a maximum thrust of 3.4 kg, and achieves a power-to-weight ratio of 2.6. The target drone uses the same motorization as the hunter drone, but is powered by a 2800-mAh three-cell LiPo battery on a YoCoo® F330 frame, and runs the ArduPilot Copter firmware on a NAVIO2 flight controller. While the hunter drone was built to be autonomous in a GPS-denied environment, the target drone was designed to be manually piloted or deployed in predetermined autonomous missions using either GPS or indoor GPS [42].

**Fig 7:**
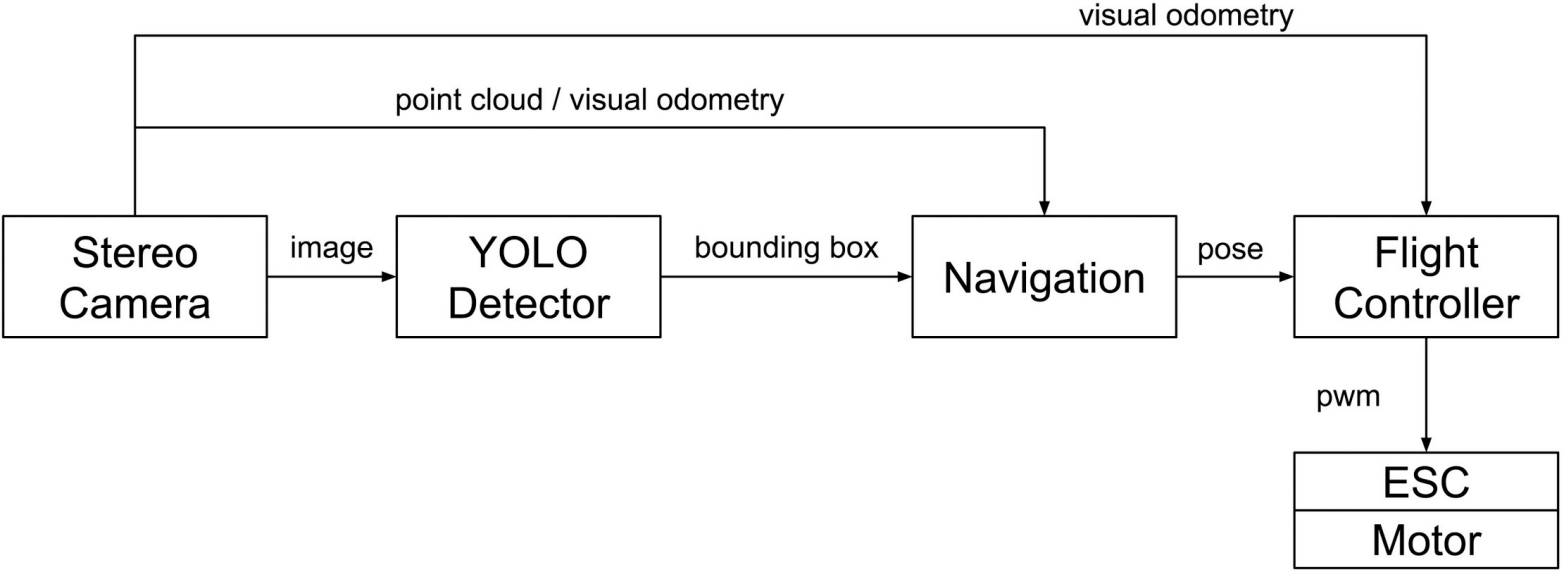
Information flow diagram for the hunter drone.

The hunter drone runs the Robot Operating System (ROS) on the Jetson TX2 and communicates via the MavLink protocol over a UART connection with HKPilot32 [43]. It uses the following five ROS nodes for operation: (1) a mavros node that communicates the set points, local position, orientation, and other flight controller parameters between the PX4 flight controller firmware and the ROS environment; (2) a stereo camera node that publishes the RGB frames, depth images, point cloud, and pose using the ZED API [41]; (3) a vision pose converter node to transform between the camera’s pose representation and the flight controller’s pose representation; (4) an AI node that extracts the bounding box information in the current RGB image; and (5) a navigation node that computes the next set point for the flight controller from the depth image, point cloud, pose, and bounding box information. This high-level control loop runs on top of the low-level control loops inside the flight controller, which stabilize the drone platform based on the inertial measurement unit, barometer, and pose information. Fig 7 shows the information flow diagram of the system.

Before conducting an autonomous mission, all ROS nodes on the drone were initiated via a secure-shell (ssh) connection to the onboard companion computer. During each mission, we streamed the camera frames and the bounding boxes of the detection algorithm to a ground-station computer. A remote pilot oversaw each experimental flight and overwrote the companion computer’s commands to control the vehicle.

## Results

### Detection algorithm

**Fig 8:**
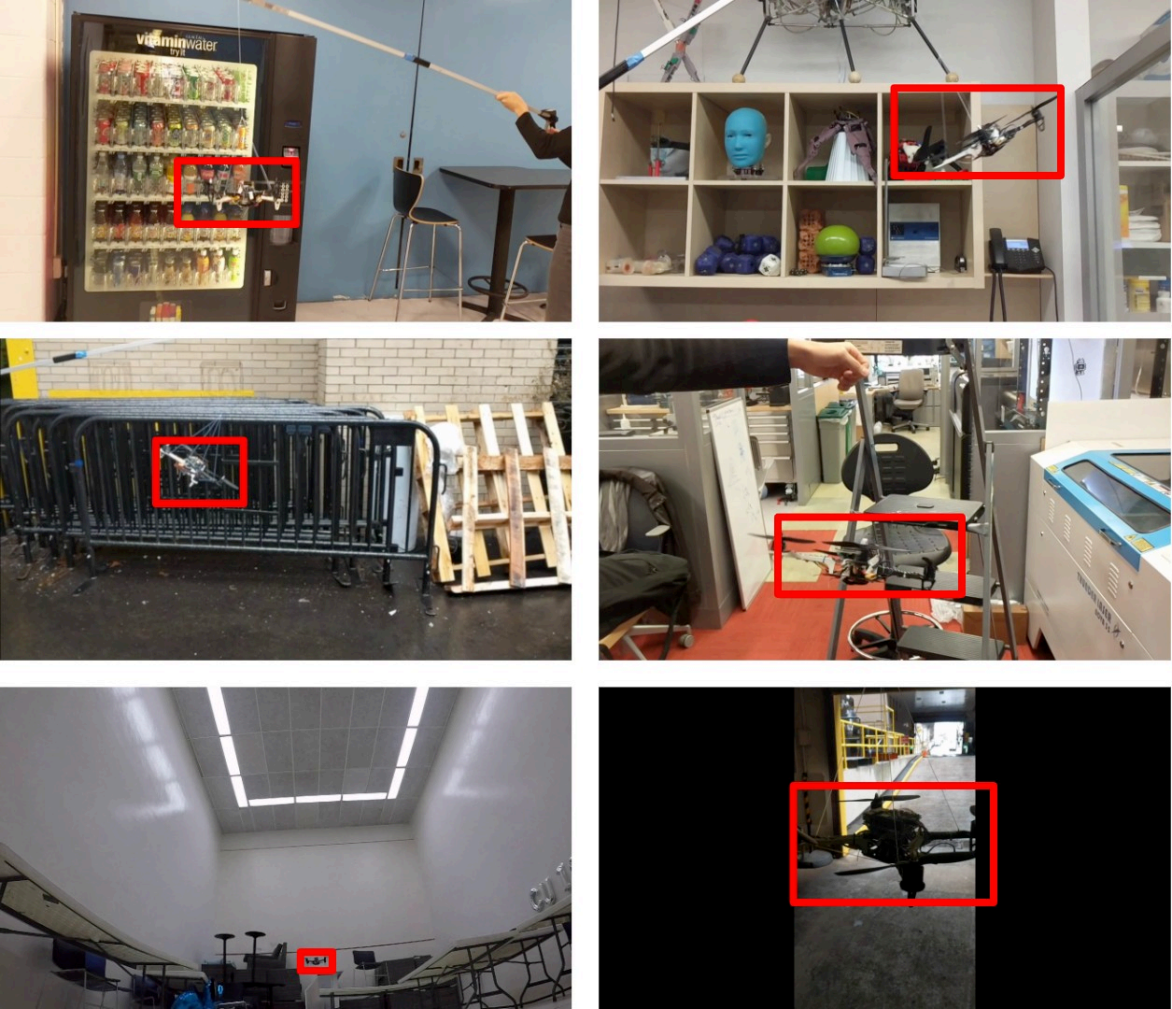
Sample images with bounding boxes obtained by the RGB model.

**Fig 9:**
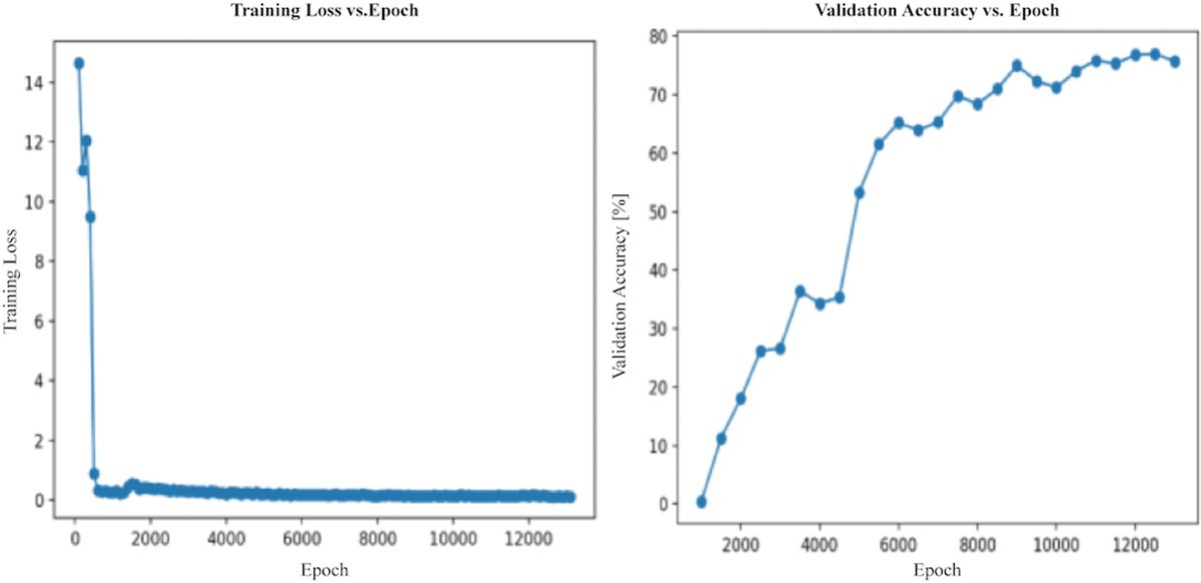
Training loss and validation accuracy graph of the RGB model. The horizontal axis represents the number of epochs in the training process. The vertical axis in the left graph shows the training/testing loss, and the vertical axis in the right graph indicates the percentage accuracy achieved on the testing data set.

On the Jetson TX2 with (256 × 256)-pixel resolution, the RGB model achieved 77% accuracy in mAP measured at 0.5 IOU with an average frame rate of 5.22 fps. Herein, intersection over union (IOU) is defined as the intersection area between the ground-truth and predicted bounding box divided by the union area of the two bounding boxes. Sample result images are shown in Fig 8, and the training loss and validation accuracy graphs are shown in Fig 9.

**Fig 10:**
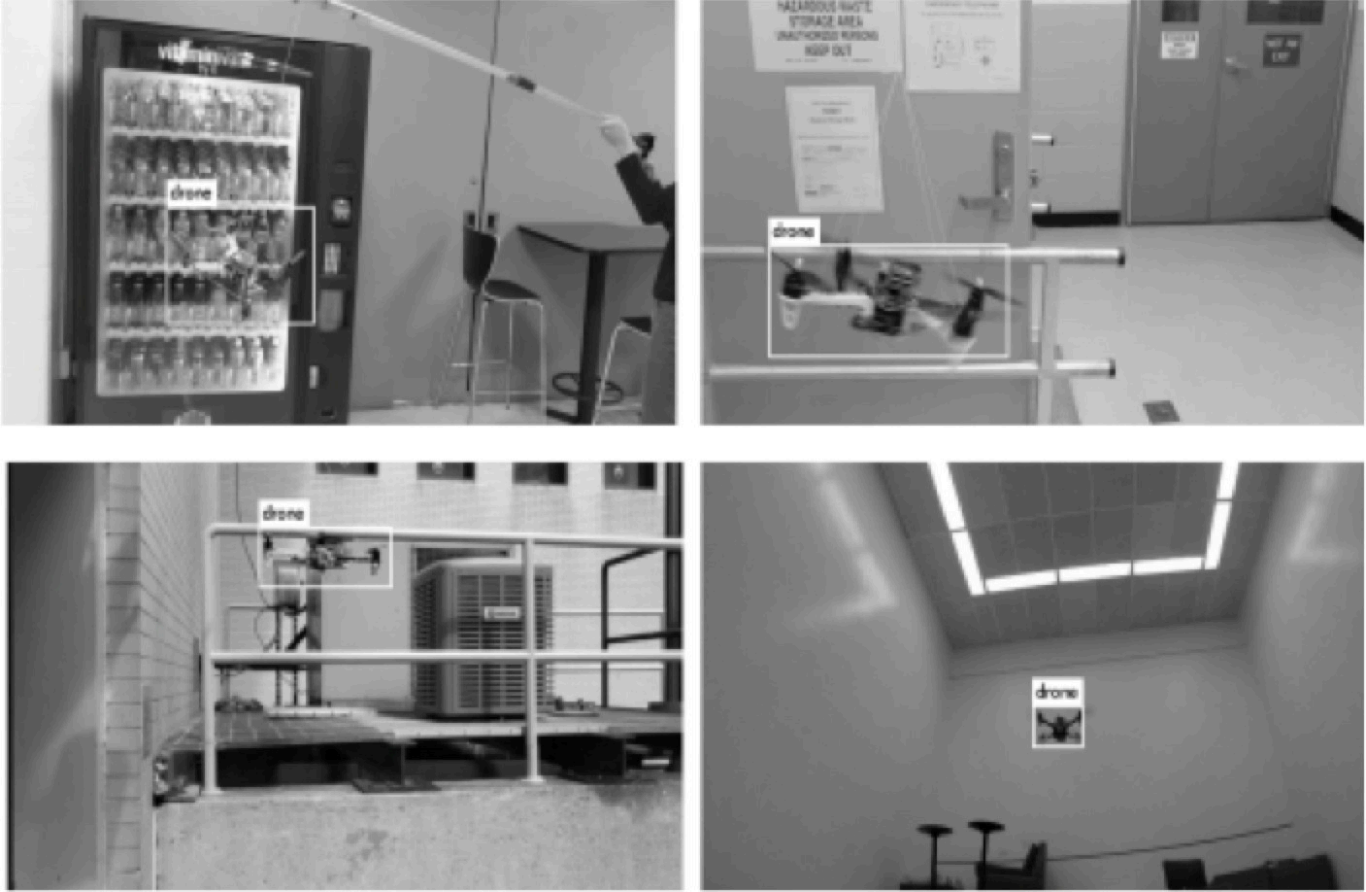
Sample images with bounding boxes obtained by the gray-scale model.

**Fig 11:**
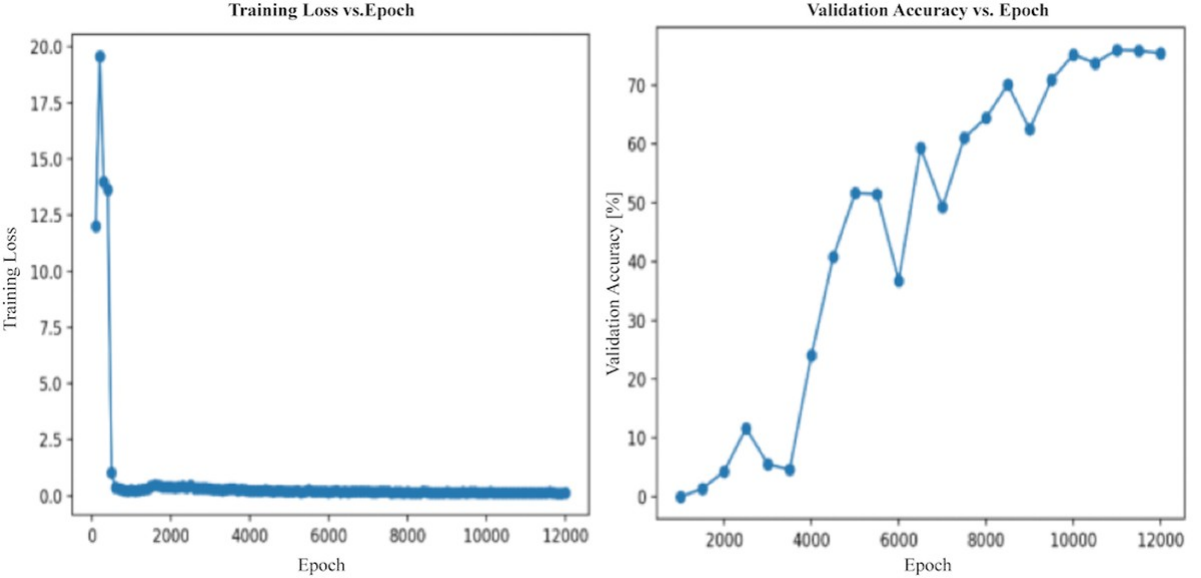
Training loss and validation accuracy graphs of the gray-scale model. The horizontal axis represents the number of epochs in the training process. The vertical axes represent the training/testing loss (left), and the percentage accuracy achieved on the testing dataset (right).

Our gray-scale model preserved the network architecture, but the number of input channels was reduced from three to one. On the Jetson TX2 with (256 × 256)-pixel resolution, this model obtained 76% accuracy in mAP measured at 0.5 IOU with an average frame rate of 8.55 fps: a 63% speed increase over the RGB model. Sample result images are shown in Fig 10, and the training loss and validation accuracy graphs are shown in Fig 11.

**Fig 12:**
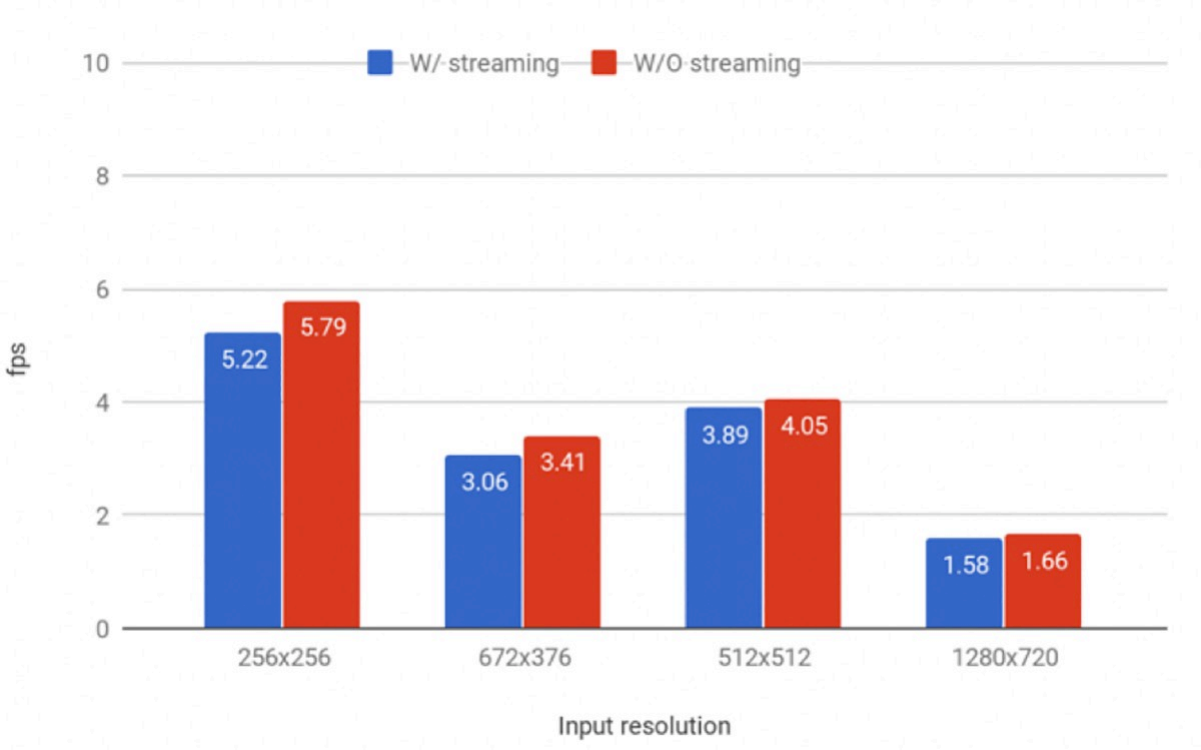
Detection speeds for the RGB model at different resolutions. Frames per second (fps) results for different image sizes produced by our trained Tiny YOLO model running on a Jetson TX2 with Darknet, with (orange) and without (blue) streaming the images to a computer. The fps was computed based on the average the classification speeds of 100 images.

**Fig 13:**
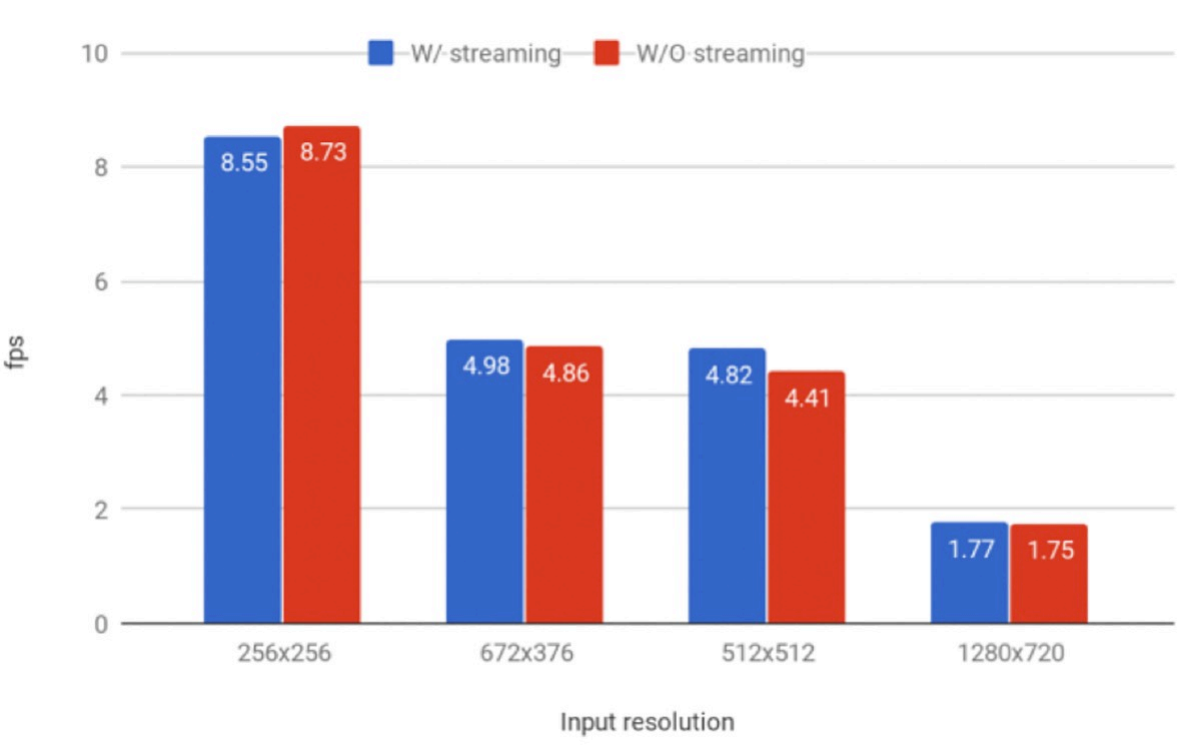
Detection speeds for the gray-scale model at different resolutions. Frames per second (fps) results for different image sizes produced by our trained Tiny YOLO model running on a Jetson TX2 with Darknet, with (orange) and without (blue) streaming the images to a computer. The fps was computed based on the average the classification speeds of 100 images.

**Fig 14:**
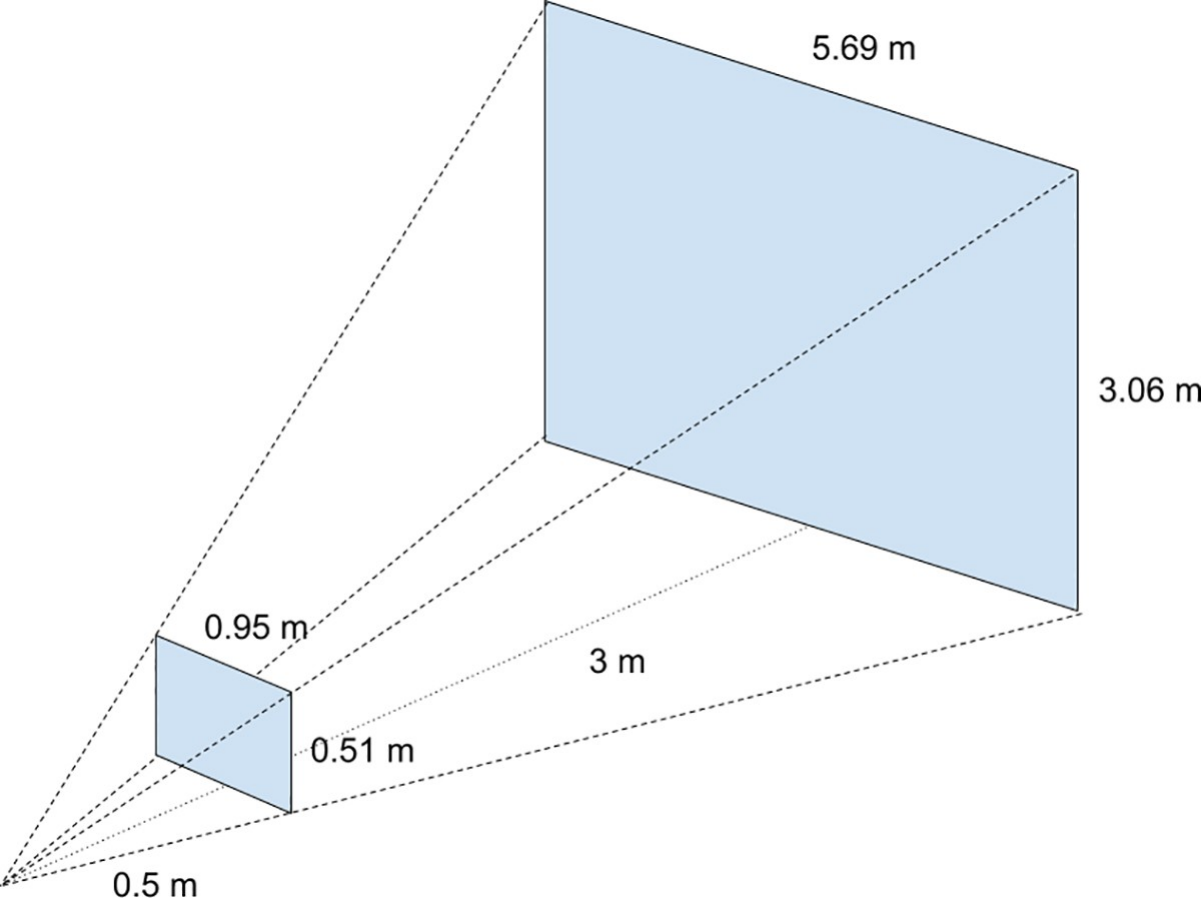
Detection range. The space between the two blue rectangles illustrates the region in which our system can detect the target drone. If the target is closer than 0.5 m to the camera or further than 3 m from the camera, the detection system fails to detect the target drone reliably.

Both models were tested at different image resolutions. Larger frame sizes slow the frame rate significantly to <5 fps and increase the risk of losing the target during the hunting process (Figs 12 and 13). The models struggled to detect drones that were more than three meters away at the system resolution of 256 × 256 pixels. Fig 14 shows the effective detection range of our perception system. The field of view of the ZED camera is dependent on the resolution and is calculated using the below formula:

$$FOV[degrees]=2\times \tan^{-1}\left(\frac{number\;of\;pixels}{2\times focal\;length\;in\;pixels}\right)*\left(\frac{180}{pi}\right)$$ (1)

where the number of pixels represents the pixel count in the image’s height or width direction as applicable. The camera manufacturer, Stereolabs provides the focal length in pixels for each resolution setting.

Furthermore, the classification speed on the Jetson TX2 was observed to be unstable, fluctuating from 5 to 10 fps. To ensure that our streaming script was not causing the slowdown, we computed the fps by averaging the classification speeds of 100 images both with and without the script running. Streaming the video did not significantly influence the classification speed of our method as shown in Figs 12 and 13. As the Jetson TX2 runs Ubuntu 16.04 (which is not a real-time operating system), the unstable detection frame rate may arise from various processes competing for computing time on both the CPU and the GPU, such as the resource-hungry ZED API.

### Flight performance

In two hunting attempts using the RGB detection model, the platform autonomously detected and chased the target drone. The goal was to follow the target drone. Our platform effectively tracked and engaged with the manually piloted target, as seen in S1 Video, which shows the second hunting attempt in full length. The hunter drone autonomously reoriented itself in the target’s direction, approaching it directly along the line of sight. Additionally, it adjusted its altitude to match that of the target, and landed autonomously near the landed target drone.

To protect our hunter platform, we limited the speed and step sizes in all directions, enabling the pilot in command to abort the mission before a crash. Because of these restrictions, the hunter drone was limited to an ascent of 40 cm, a maximal rotation of 30°, and a forward motion of 10 cm per navigation-control cycle. The rate of the control cycle is limited by the speed of the classifier at ~5 cycles per second. As a result, when the target drone moved erratically, the hunter could not keep up and would return to scanning for the target.

We determined three reasons for the hunter losing its target: erratic movement by the target drone and false positives and false-negatives by the detection algorithm. From our flight footage, we can conclude that the detection algorithm performs worse when the black target drone has poor contrast with its background or when other complex shapes are present in the frame. Over the two missions, for 25.5 s out of a total of 113 s the hunter either did not recognize the target or misclassified another object as the target; this is in alignment with our detection accuracy of 77% of the RGB algorithm used. In the case of a false-positive, the hunter drone approaches the object falsely classified as a drone. However, the hunter can recover the target drone if it remains in its field of view. In case of a false-negative, the drone will resume its search pattern to look for the target.

Both the VIO localization and the tracking algorithm were sensitive to poor lighting conditions. Experiments conducted in poor lighting conditions were twice as likely to lead to instability, drifting, or crash. Similarly, the walls in our testing environment would lead to large error accumulation in the VIO localization algorithm. In the first of the two hunting attempts, error accumulation lead to severe instability and forced an early abortion after only 25 s of flight time.

## Discussion

Drone hunting in GPS deprived environments is a robotic challenge that integrates high-speed autonomous navigation, object detection and tracking, obstacle avoidance, friend or foe identification, and new sensor technologies to achieve a single goal. Advancements in drone hunting technology benefit other fields, such as search and rescue. Thus, in this study, we present a first approach to drone hunting.

Our current hunter drone prototype is too heavy for aggressive flight maneuvers, and it lacks the computational resources for faster tracking. Further improvements in camera technology and compute modules should increase the computational power of our platform while reducing its weight. For example, the Jetson AGX Xavier compute module promises multiple times better performance than our currently used Jetson TX2[44]. Additionally, switching from the ZED camera to the Intel RealSense R435 camera would reduce weight and provide more reliable depth information.

Building a viable real-world solution is a complex proposition that will require more than just the above-mentioned additions. First, significantly larger datasets will be needed to enable hunters to detect different types of drones. The detection algorithm will also need to be able to cope with detecting targets in cluttered environments and be resilient to occlusion and lighting changes. Further, it will need to be paired with a robust tracking algorithm that can learn the targets signature and thereby recognize it among other drones to avoid false positives. Additional sensors, such as directional microphones, could also be added to allow the hunter to identify the direction in which the target escaped after leaving its camera’s field of vision. Finally, a suitable takedown mechanism must be selected that does not endanger bystanders. Creating a self-sufficient platform drone platform that satisfies all these requirements will be difficult.

Unsurprisingly, state-of-the-art commercial solutions, such as the Airspace Interceptor, rely on sophisticated ground stations for computation and large UAVs with a net guns for target apprehension [17]. For this study, we chose a particularly challenging scenario that demands GPS-denied operation and onboard computation without relying on a downlink. Common real-world drone hunting applications, such as preventing espionage on corporate properties, countering smuggling operations, or policing airspace violations around airports, do not typically take place in GPS-denied environments or prohibit the use of ground-station downlinks. In these and similar scenarios, being able to rely on GPS and offload computationally intensive tasks to a ground station to take advantage of high-performance computational resources eases the drone hunting task. A downlink would also allow the operator to make a friend-or-foe decisions on-the-fly if the hunter detects multiple drones and is uncertain which to target, a task that is very challenging to automate.

Potential future drone hunting advances are not limited to the above improvements, but could also include exploring alternative sensor combinations. For example, the hunter platform could be made more effective by adding sensors that track the target’s RF signal or using directional microphones to determine its direction. Fast object localization and tracking algorithms, such as that proposed by Wu et al. have the potential to improve hunting performance [45]. Their CPU-optimized algorithm was able to achieve an average frame rate of 141 fps on a desktop CPU. In addition, since hunting drones have limited onboard computational resources, offloading repetitive tasks, such as VIO or Simultaneous Localization and Mapping (SLAM) to a dedicated FPGA chip could ease the load on their primary CPUs and free up resources for the tracking algorithm[46,47].

We also believe that these technological developments will make drone hunting more challenging, as targets will attain higher levels of autonomy on smaller, faster, and more agile platforms. This means hunter drones will also have to improve to remain dominant. Further, we believe that drone hunting’s long-term future does not lie with large platforms, such as the Airspace Interceptor, but rather with small, agile, autonomous platforms that can collaborate in counter-UAV operations.

## Conclusion

We applied a deep-learning-based approach to autonomous drone hunting by a small UAV in a GPS-denied environment. Our approach was developed without a motion-capturing system and introduced a baseline approach for autonomous drone hunting in a GPS-denied environment. The detection accuracy of our present system is 77%. The detection speed, 8.33 fps, is bottlenecked by the limited computing power. Advanced compute modules are expected to improve the frame rates and response times of such systems. Our approach creates a baseline for further development in drone hunting by the research community. In this vain, we share not only our findings, but also the code and data needed to reproduce them in OSF project repository here https://osf.io/n9q78/. Improving the tracking speed and accuracy, inferring the location of the target when occluded, and adding a tracking algorithm that can discriminate between multiple possible targets are future design challenges.

## Supporting information

S1 Video Full length drone hunting video. (MP4)
